# Kisspeptin Modulates Luteinizing Hormone Release and Ovarian Follicular Dynamics in Pre-pubertal and Adult Murrah Buffaloes

**DOI:** 10.3389/fvets.2018.00149

**Published:** 2018-07-04

**Authors:** Vishalkumar Pottapenjera, Srinivasa R. Rajanala, Chandrasekhar Reddy, Arunakumari Gangineni, Kiran Avula, Sandeep K. Bejjanki, Sriravali Sathagopam, Surabhi Kesharwani, Sathya Velmurugan

**Affiliations:** ^1^Department of Veterinary Gynaecology and Obstetrics, College of Veterinary Science, PV Narasimha Rao Telangana Veterinary University, Hyderabad, India; ^2^National Institute of Animal Biotechnology, Hyderabad, India

**Keywords:** kisspeptin, buserelin, Murrah buffaloes, LH, FSH, follicular dynamics, oestrous synchronization

## Abstract

Kisspeptin is a neuropeptide that governs the reproductive axis upstream to GnRH. We wanted to study whether kisspeptin modulates plasma LH and FSH levels and ovarian follicular dynamics in buffaloes and whether kisspeptin can be used for fixed time artificial insemination (FTAI). We carried out these studies in comparison with buserelin, a potent GnRH agonist. Kisspeptin dose-dependently increased plasma LH levels. However, the kisspeptin-induced increase in LH was short-lived as the peak reached in 15–30 min returned to basal values by 1–2 h. The kisspeptin-induced increase in LH level was less compared to buserelin-induced increase in LH level which sustained over time. Kisspeptin did not enhance FSH release while buserelin resulted in a gradual increase over time. LH response to repeated injections of kisspeptin was greater than that induced by buserelin. While buserelin induced an increase in the number of follicles, kisspeptin induced an increase in the growth rate of the follicle. In adult cycling animals, while both the drugs increased plasma LH levels, the increase was greater in buserelin group compared to kisspeptin group. In contrast to the findings in pre-pubertal animals, kisspeptin induced an increase in both the number as well as the size of follicles compared to buserelin. Our studies on oestrus synchronization, using either kisspeptin-PGF_2α_-kisspeptin protocol or buserelin-PGF_2α_-buserelin Ovsynch protocol on day 0, 7, and 9, respectively, revealed that kisspeptin increased the number of follicles at wave emergence and the diameter of dominant follicle after 2nd dose of drug, the oestrus response rate and duration of oestrus, compared to buserelin. However, conception rate was not significantly different among the groups. From our studies, it appears that Kp and Buserelin differentially modulate follicular dynamics depending on the reproductive age of the animals.However, studies in a larger herd are required to confirm whether kisspeptin can be used for oestrous synchronization in buffaloes.

## Introduction

Kisspeptin is one of the controllers of hypothalamo-pituitary-gonadal (HPG) axis upstream to gonadotropin releasing hormone (GnRH). Neuroendocrine control of GnRH neurons by kisspeptin orchestrates the sequences that take place during the oestrous cycle (1). In the hypothalamus, kisspeptin is expressed in the arcuate nucleus and rostral periventricular region in rats and in the arcuate nucleus and preoptic area in sheep (2, 3). Apart from hypothalamus, expressionof *Kiss1* gene and immunoreactivity of kisspeptin and its receptor, GPR54, have been reported in extra-hypothalamic tissues, such as ovaries, in rats and sheep (4).

Currently, GnRH or luteinizing hormone (LH) are being used to induce ovulation, via oestrus synchronization protocol, in subfertile buffaloes. However, the conception rate is only 50–55% in a synchronization protocol that uses GnRH and prostaglandin (5). Hence, new therapeutic strategies aiming at increased conception rate and reduced fertility disorders are warranted. Studying the endocrine profile and follicular dynamics upon kisspeptin administration would be a key step toward kisspeptin-based therapeutic strategies.

Induction of LH secretion by kisspeptin administration has been reported in steer, pre-pubertal calves and ovariectomized cows (6). In buffaloes, intramuscular injection of kisspeptin has been shown to induce LH secretion in both breeding and non-breeding seasons (7). Though kisspeptin has been proposed as a regulator of ovulation at the ovarian level, the effect of kisspeptin on the follicular dynamics as a whole has not been studied yet. In order to address this, we studied the endocrine profile and follicular dynamics upon kisspeptin administration in pre-pubertal and adult buffaloes with the specific hypothesis that kisspeptin regulates follicular dynamics in buffaloes. We also report our findings on oestrus synchronization in buffaloes when kisspeptin is used instead of buserelin, a potent GnRH agonist.

## Materials and methods

### Experimental animals

All animals were maintained at the Department of Instructional Livestock Farm Complex, College of Veterinary Science, PV Narasimha Rao Telangana Veterinary University (PVNR TVU), Hyderabad, India. The experiments were approved by the Institutional Animal Ethics Committee of College of Veterinary Science, PVNR TVU.

Six pre-pubertal Murrah buffaloes, procured from Yeluru village in Medak district, Telangana, and five pre-pubertal Murrah buffaloes, born at the Farm Complex, were quarantined for 2 weeks and acclimatized for experimental sheds. These animals weighed 200–250 kg and were between 1 and 1.5 years of age, approximately. Throughout the experimental period (i.e., March to May; summer), the heifers were maintained as a group and were housed using semi-intensive system, which included a covered shed and an open paddock, under identical conditions of concentrate and roughage feeding and other managemental practices.

Twelve multiparous cycling non-pregnant lactating Murrah buffaloes, aged between 4 and 10 years, all in their mid-lactation, with parity ranging from 2 to 5, from the Farm Complex, were used for studies in adult buffaloes. All the animals were clinically normal and had regular oestrous cycles. During the experimental period (i.e., August to February; autumn and winter), these adult buffaloes were maintained under semi-intensive housing system and were fed with green fodder, concentrate feed, and *ad-libitum* drinking water.

### Drugs

Bovine kisspeptin (Tyr-Asn-Trp-Asn-Ser-Phe-Gly-Leu-Arg-Tyr-NH2), a decapeptide, was commercially synthesized (Biotech Desk). Stock solutions were prepared at the concentration of 5 mg/ml in distilled water and aliquots were frozen at −20°C until use. Stocks were diluted in normal saline to a working solution of 2.5 ml volume. Buserelin acetate (Receptal Vet; Intervet India—MSD Animal Health) was commercially obtained. Each ml of the commercial preparation contained 4.2 μg of buserelin acetate equivalent to 4.0 μg of buserelin. Drugs were administered either intramuscularly (IM) or intravenously (IV). We chose 10 μg/animal as the dose rate for buserelin as recommended by the manufacturer for delayed ovulation and anovulation. For kisspeptin, we used doses starting from 5 μg/kg as this has been shown to significantly increase plasma LH in cattle (8).

### ELISA

Plasma LH and FSH concentrations were quantified using bovine ELISA kits (Abnova; Catalogue No. KA2280 and KA2278) as per the manufacturer's instructions. The limits of detection were 0.25 and 0.5 ng/ml, respectively, for LH and FSH. The samples were analyzed in duplicates. The inter-assay coefficient of variability (CV) was less than 12.5% and the intra-assay CV was less than 7.5%.

### Studies in pre-pubertal buffaloes

#### Dose-response study

**Figure 1:**
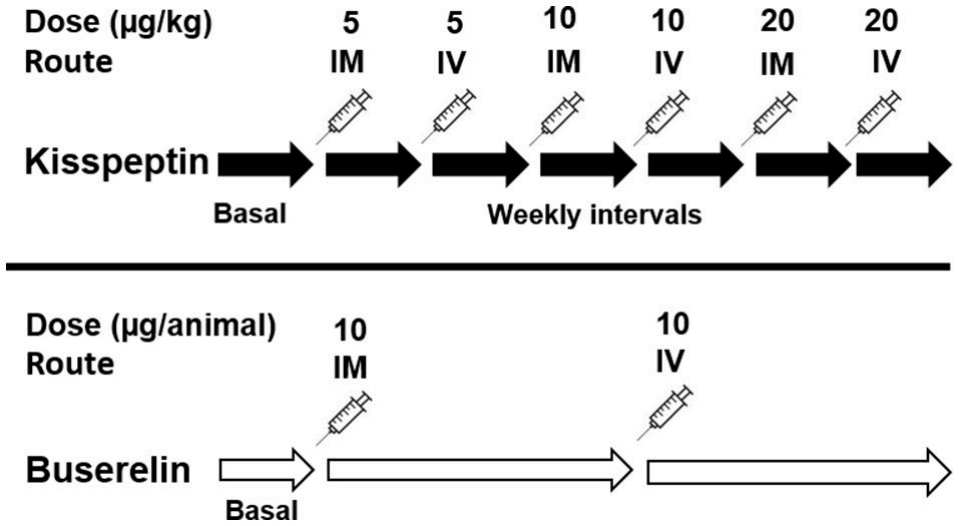
Schematic representation of methodology for dose-response study. Following initial basal observation of follicular dynamics for a week, kisspeptin was administered at three different doses at two routes (i.e., IM or IV) at weekly intervals approximately. A minimum of 4 ultrasound observations of follicular dynamics were carried out on alternate days between two injections, as depicted by the solid black arrow. Buserelin was administered at a single dose rate recommended by the manufacturer via two routes (i.e., IM or IV) at triweekly intervals approximately, as depicted by the longer white arrows.

In pre-pubertal buffaloes, dose-response studies on the effect of kisspeptin (bolus injections at 5, 10, and 20 μg/kg body weight; or 3.85, 7.7, and 15.4 nmol/kg body weight; 2.5 ml; IM and IV) on plasma LH concentration was carried out (*n* = 6; kisspeptin group). In another group (*n* = 5; buserelin group), LH profiles following administration of buserelin at the recommended dosage (10 μg/animal, 2.5 ml, IM and IV) were studied for comparison. Plasma follicle stimulating hormone (FSH) was also estimated upon administration of buserelin and the maximal dose of kisspeptin (i.e., 20 μg/kg). In kisspeptin group, following initial basal observation of follicular dynamics for a week, kisspeptin was administered at the above doses at weekly intervals, i.e., each dose was administered at regular interval in such a way that a minimum of 4 ultrasound observations of follicular dynamics could be carried out on alternate days. The methodology is schematically given in Figure 1. On the day of administration of drugs, the buffaloes were fitted with intravenous cannula in jugular vein enabling sequential sampling. The time points of sampling following IM kisspeptin/buserelin are: 30 min, 1, 2, 3, and 4 h. The same for IV kisspeptin/buserelin are: 15, 30 min, 1, 1.5, and 2 h. The delay in collecting blood samples, if any, was never more than 2 min of the prescribed time point. Blood samples, collected separately in heparinized vials and serum tubes, were kept on ice until reaching the lab; plasma and serum were separated and stored at −20°C until LH and FSH levels were analyzed using bovine ELISA kits (Abnova).

#### Effect of repeated injections of kisspeptin on plasma LH and FSH

In order to mimic a long-acting agonist or a sustained release system, and to study whether GPR54 is desensitized upon multiple injections of kisspeptin, the drug was administered IV at 10 μg/kg at 30 min intervals for five times and the levels of FSH and LH were studied. Three pre-pubertal buffaloes, in which LH response to kisspeptin was consistent with dose and was the highest, were chosen for this study.

#### Follicular dynamics

The pre-pubertal buffalo heifers were monitored for their ovarian follicular growth and ovulation, if any, on every alternate day by ultrasound examination using a real-time B mode ultrasound machine (Aloka ProSound 2, Hitachi) equipped with a 5–7.5 MHz linear array transducer designed for per-rectal examination. The transducer was placed on the ovary through rectal wall and scanning was accomplished in several planes to identify follicles. The desired images were frozen on the screen and measurements were taken using an inbuilt calibration system. The number and size of the follicles were recorded. The growth of individual follicles was tracked with ultrasound examination every other day. Average number of follicles per wave was calculated by summing up the follicular count for each day of the wave and dividing by the duration of the wave. Total number of follicles at wave emergence represents the number of follicles observed on the day of wave emergence which occurs after the dominant follicle of the previous wave starts to become atretic. The smallest diameter considered for identification of follicles was 2 mm. Follicles were categorized as small (<5 mm), medium (5–8 mm) and large follicles [>9 mm; (9)]. Growth rate was calculated for all the growing follicles. Before beginning any of the experiments, follicular dynamics was studied for a duration of 10 days without administration of any drug and this was regarded as the control period. Ovulation was not observed in any of the pre-pubertal heifers during the entire period of the study.

### Studies in adult buffaloes

#### Effect of kisspeptin vs. buserelin on plasma LH and FSH and on follicular dynamics

During a preliminary study, ultrasound examinations were carried out daily for two consecutive oestrous cycles to know the follicular wave pattern in all experimental animals (*n* = 12). It was observed that out of 12 buffaloes 10 showed two-wave pattern and two buffaloes showed three-wave pattern. After ascertaining the follicular wave pattern, experimental buffaloes were randomly divided into two groups and administered either kisspeptin (20 μg/kg, IV; *n* = 6) or buserelin (10 μg, IV; *n* = 6) on 11th or 8th day of estrous cycle depending on whether the oestrous cycle is of two-wave or three-wave pattern, respectively, correlating with emergence of waves that lead to ovulation. Blood samples were collected before (basal) and after (at the expected peak time point as seen from pre-pubertal data) the drug administration. Following the administration of kisspeptin or buserelin, the animals were examined daily for 25 days using ultrasound for follicular dynamics.

#### Effect of two different protocols involving kisspeptin or buserelin on follicular dynamics during oestrus synchronization

**Figure 2:**
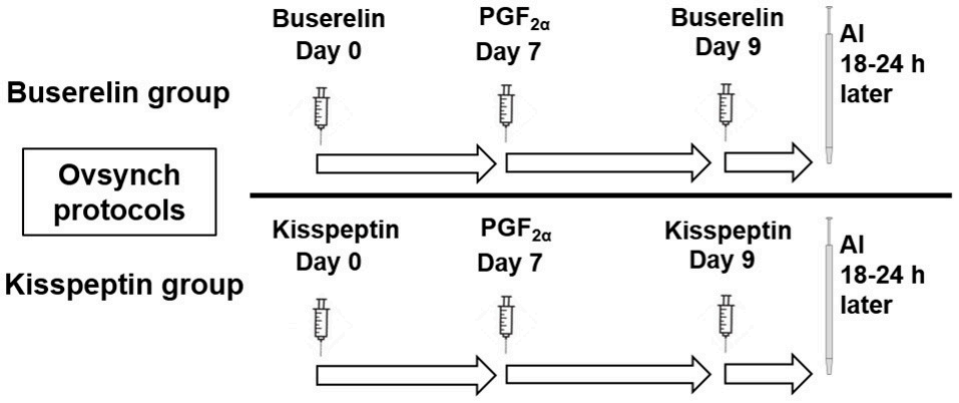
Schematic representation of Ovsynch protocols tested in Murrah buffaloes for oestrus synchronization using either buserelin or kisspeptin. On day 0 and 9, animals in buserelin group were treated with buserelin (10 μg/animal, IV) while that in kisspeptin group received kisspeptin (20 μg/kg, IV). PGF_2α_ (500 μg, IV) was administered on day 7. Fixed time AI was done 18–24 h post second dose of buserelin or kisspeptin.

Two months after studying the effect of drugs on follicular dynamics, the efficacy of kisspeptin on synchronization of oestrus in buffaloes was studied in comparison with buserelin. The buffaloes treated with buserelin in the earlier study were subjected to Ovsynch protocol that involved administration of buserelin (10 μg, IV) on day 0, followed by PGF_2α_ (500 μg Cloprostenol sodium, Vetmate, Vet Care) on day 7 and a second injection of buserelin (10 μg, IV) on day 9 (Figure 2). The buffaloes treated with kisspeptin in the earlier study were administered kisspeptin (20 μg/kg, IV) following the same protocol. The first dose was administered on a day that was randomly chosen. Fixed-time artificial inseminations (FTAI) were done in both the groups 18-24 h after the administration of second dose of kisspeptin or buserelin. Ovarian ultrasonography was carried out daily from the day of initiation of the synchronization protocol until insemination to study the time of wave emergence after first dose of the drug, number of follicles at wave emergence, diameter of the dominant follicle after the administration of first and second dose of kisspeptin or buserelin. Oestrus response rate and duration of oestrus were recorded by visual inspection. First service conception was recorded by diagnosing pregnancy by trans-rectal examination on day 45.

### Statistics

Data were compiled using Excel (Microsoft Inc) and given as mean ± SEM.Tthe data were normally distributed and variances were homogeneous; no manipulations or transformations were undertaken. GraphPad Prism (Version 6.07, GraphPad Software Inc.) statistical software was used to analyze endocrine profile data using two-way repeated measures analysis of variance (RM ANOVA) followed by Dunnett's or Sidak's multiple comparison tests. SPSS (Version 16.0, SPSS Inc) statistical software was used to analyze follicular dynamics data using *t*-test or ANOVA followed by Duncan's multiple range test. Differences were considered to be significant when *P* < 0.05.

## Results

### Dose-response study in pre-pubertal buffaloes

**Figure 3:**
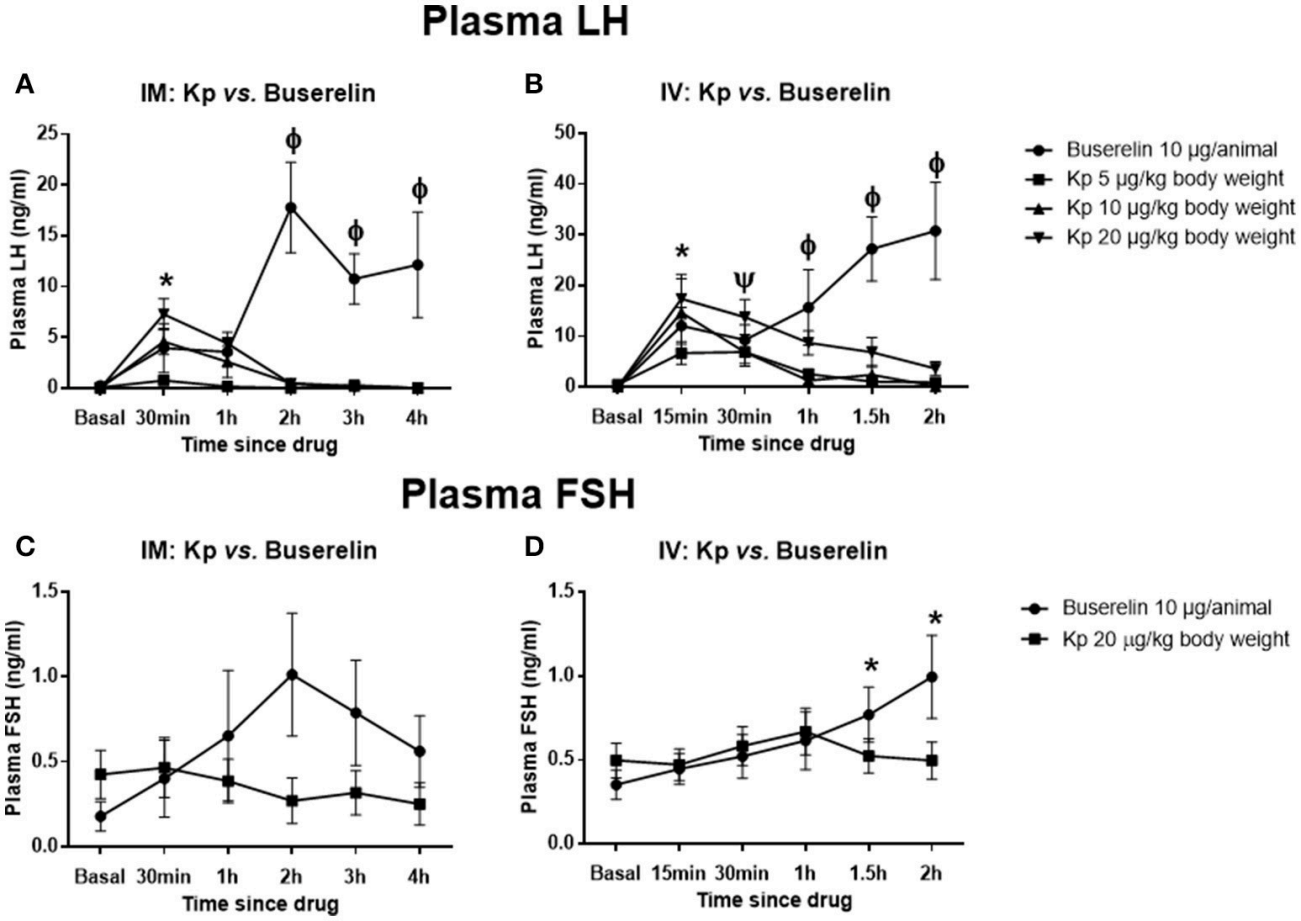
Effect of kisspeptin or buserelin on plasma LH and FSH levels in pre-pubertal buffaloes out of the breeding season. **(A)** Kisspeptin IM dose-dependently elevated plasma LH levels in pre-pubertal buffaloes. However, the LH level reached basal values by 2 h. Buserelin resulted in a gradual but sustained increase in LH level (*kisspeptin 20 μg/kg: basal vs. at 30 min; kisspeptin: 5 vs. 20 μg/kg at 30 min; ϕbuserelin: basal vs. at 2, 3, and 4 h; buserelin vs. all kisspeptin groups at 2, 3, and 4 h time points; *P* < 0.05, RM ANOVA followed by Dunnett's multiple comparison test). **(B)** The release kinetics of LH upon IV injections of the drugs were similar to that of IM injections of drugs. (*buserelin: basal vs. at 15 min; kisspeptin: basal vs. kisspeptin 10 and 20 μg/kg at 15 min; Ψkisspeptin 20 μg/kg: basal vs. at 30 min; ϕbuserelin: basal vs. at 1, 1.5, and 2 h; buserelin vs. kisspeptin 5 and 10 μg/kg at 1 h; buserelin vs. kisspeptin 5, 10, and 20 μg/kg at 1.5 and 2 h; *P* < 0.05, RM ANOVA followed by Dunnett's multiple comparison test). **(C)** There was no significant increase in plasma FSH upon IM injections of both drugs. **(D)** Buserelin significantly elevated plasma FSH at 1.5 and 2 h post-injection, however, it was not significantly different from kisspeptin group (*buserelin: basal vs. at 1.5 and 2 h; *P* < 0.05, RM ANOVA followed by Dunnett's multiple comparison test). Data represented as mean ± SEM (*n* = 5–6 animals at each time point).

Kisspeptin (IM and IV) dose-dependently elevated plasma LH levels in pre-pubertal buffaloes (IM: kisspeptin 5 vs. 20 μg/kg at 30 min, *P* < 0.05; IV: kisspeptin 5 vs. 10 and 20 μg/kg at 15 min, *P* < 0.05; two-way RM ANOVA, followed by multiple comparison tests; Figures 3A,B). The peak values were observed at 15 and 30 min time points for IV and IM injections, respectively. However, the LH level reached basal values by 2 h. Upon administration of buserelin (IM and IV), there was a gradual and sustained release of LH (IM: Basal vs. 2, 3, and 4 h post-drug, *P* < 0.05; IV: Basal vs. 1, 1.5, and 2 h post-drug, *P* < 0.05; two-way RM ANOVA, followed by multiple comparison tests; Figures 3A,B). While IM administration of kisspeptin (20 μg/kg) maximally elevated the LH concentration only to 7.29 ± 1.7 ng/ml (at 30 min), IV injection elevated the same to 17.4 ± 4.4 ng/ml (at 15 min; *P* = 0.04, *t*-test). Though IV injection of buserelin increased the LH concentration at 2 h post-drug to 30.79 ± 11.47 ng/ml, it was not significantly higher than the concentration at 2 h (17.81 ± 5.16 ng/ml) after IM injection. FSH was not significantly elevated upon kisspeptin, while buserelin IV increased FSH in a slow and sustained manner (IV: Basal *vs*. at 1.5 and 2 h, *P* < 0.05, RM ANOVA followed by multiple comparison tests; Figures 3C,D), similar to that of LH response. The interaction between time and treatment was significant for both routes of injections and for both FSH and LH (*P* < 0.001, two-way RM ANOVA).

### Follicular dynamics in pre-pubertal buffaloes

We observed follicular wave pattern in pre-pubertal heifers with each wave lasting for about 8–8.5 days, without any ovulation. Emergence of a wave was observed when the dominant follicle of the previous wave started to become atretic. The data were combined for different doses and/or routes of the drugs as and when there were no significant differences. The average number of follicles in a wave and the number of follicles at wave emergence were significantly more in buserelin group compared to kisspeptin group and control (*P* < 0.05, one-way ANOVA followed by Duncan multiple range test). Though the overall growth rate of follicles was increased upon administration of both the drugs, kisspeptin at 5 μg/kg, IM, significantly increased the growth rate of the follicles compared to all other doses of kisspeptin and of buserelin (*P* < 0.05, one-way ANOVA followed by Duncan multiple range test). The maximum diameter of dominant follicle was not different among the groups. The results are summarized in Table 1.

**Table 1:** Comparison of ovarian response to kisspeptin or buserelin in pre-pubertal buffaloes out of the breeding season.

**S. No**.	**Parameters**	**Control (*n* = 11)**	**Kisspeptin (*n* = 6)**	**Buserelin (*n* = 5)**
1.	Length of wave (d)	8.2 ± 0.4	8.0 ± 0.4	8.5 ± 0.5
2.	Average number of follicles per wave	3.49 ± 0.09^b^	3.46 ± 0.05^b^	4.12 ± 0.06^a^
	Small follicles (<5 mm)	1.89 ± 0.08^b^	1.81 ± 0.05^b^	2.13 ± 0.06^a^
	Medium Follicle (5–8 mm)	1.39 ± 0.05^b^	1.60 ± 0.03^a^	1.63 ± 0.03^a^
	Large follicles (>8 mm)	1.06 ± 0.03^a^	1.59 ± 0.03^b^	1.18 ± 0.02^a^
3.	No. of follicles at wave emergence	4.57 ± 0.20^b^	4.67 ± 0.10^b^	5.90 ± 0.11^a^
4.	Growth rate of follicle (mm/d): Overall (doses and routes combined)	0.37 ± 0.04^a^	0.66 ± 0.05^b^	0.59 ± 0.10^b^
5.	Effect of IM administration of drugs on follicular growth rate (mm/d)			
	5 μg/kg BW	0.37 ± 0.04^a^	0.80 ± 0.17^c^	0.57 ± 0.19^b^
	10 μg/kg BW		0.57 ± 0.09^b^	
	20 μg/kg BW		0.67 ± 0.08^b^	
6.	Effect of IV administration of drugs follicular growth rate (mm/d)			
	5 μg/kg BW		0.60 ± 0.13^b^	0.6 ± 0.13^b^
	10 μg/kg BW	0.37 ± 0.04	0.65 ± 0.15^b^	
	20 μg/kg BW		0.70 ± 0.19^b^	
7.	Maximum diameter of dominant follicle (mm)	10.5 ± 0.48	10.81 ± 0.19	10.76 ± 0.25

*Kisspeptin was administered at three different doses (5, 10, and 20 μg/kg) at two routes (i.e., IM or IV) at weekly intervals approximately. A minimum of 4 ultrasound observations of follicular dynamics were carried out on alternate days between two injections. Buserelin was administered at a single dose rate (10 μg/animal) recommended by the manufacturer via two routes (i.e., IM or IV) at triweekly intervals approximately. Follicular growth rate was the greatest when kisspeptin was administered at 5 μg/kg, IM. Numerical values with different superscripts within the rows are significantly different. One way ANOVA followed by Duncan's multiple range test (p < 0.05)*.

### Effect of repeated administration of kisspeptin on plasma LH and FSH

**Figure 4:**
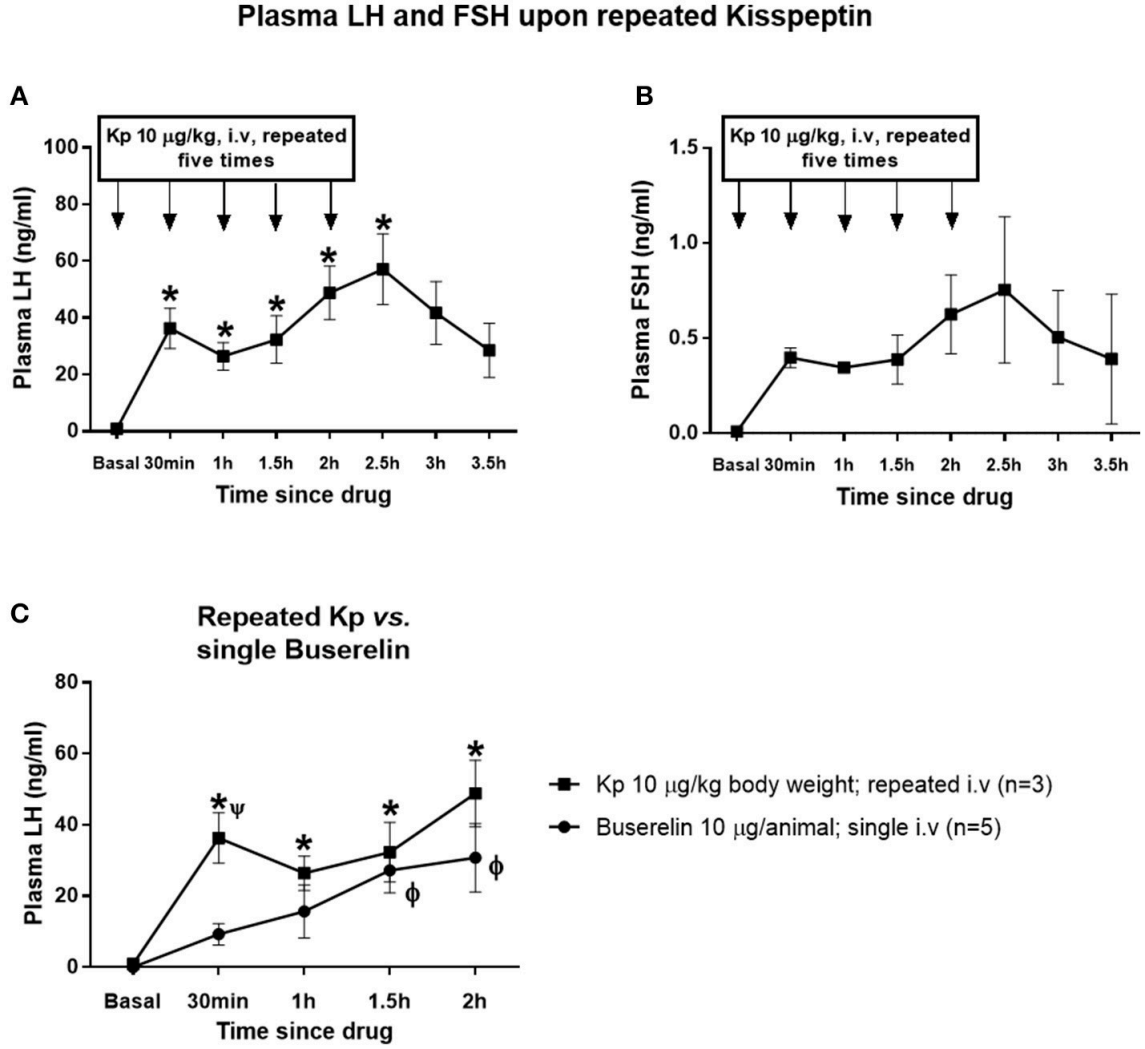
Effect of repeated administration of kisspeptin on plasma LH and FSH. **(A)** Repeated administration of kisspeptin (10 μg/kg) elevated LH in a sustained manner (*vs. basal; *P* < 0.05, one-way RM ANOVA). **(B)** There was no significant increase in FSH level upon repeated administration of kisspeptin. **(C)** LH levels upon repeated kisspeptin administration were higher than that upon a single injection of buserelin (*kisspeptin: basal vs. all time points; ϕ buserelin: basal vs. 1.5 and 2 h; Ψ buserelin vs. kisspeptin at 30 min; RM ANOVA followed by Dunnett's and Sidak's multiple comparison test). Data represented as mean ± SEM (*n* = 3–5 animals at each time point).

Repeated administration of kisspeptin (10 μg/kg, IV, a total of five injections at 30 min intervals) elevated LH in a sustained manner (Figure 4A). Compared to basal concentration, the LH level remained significantly elevated until 30 min after repeated kisspeptin (*P* < 0.05, one-way RM ANOVA followed by Dunnett's multiple comparison tests). After increasing from basal level to 36.28 ± 8.66 ng/ml upon first kisspeptin injection, the LH concentration reached 26.42 ± 5.93 ng/ml upon second injection. However, compared to basal concentration, LH level increased further upon third and fourth injections to 32.32 ± 10.28 and 48.81 ± 11.49 ng/ml, respectively. At 2.5 h, i.e., 30 min after the fifth kisspeptin injection, LH concentration reached a maximum of 57.15 ± 15.27 ng/ml, after which it started declining. Though the FSH concentration followed a similar pattern, it was not significant (Figure 4B). LH levels upon repeated kisspeptin administration was comparable with the response following buserelin (10 μg; IV) in terms of sustained release of LH (Figure 4C). Though LH level upon repeated-kisspeptin remained elevated throughout the observation, without being statistically significant, compared to that upon single injection of buserelin, at 30 min, kisspeptin-induced increase in LH concentration was significantly higher than buserelin-induced LH (*P* < 0.05, two-way RM ANOVA followed by Dunnett's multiple comparison tests). However, there was no interaction between time and treatments (*P* > 0.05, two-way RM ANOVA).

### Effect of kisspeptin vs. buserelin on plasma LH and FSH in adult buffaloes

**Figure 5:**
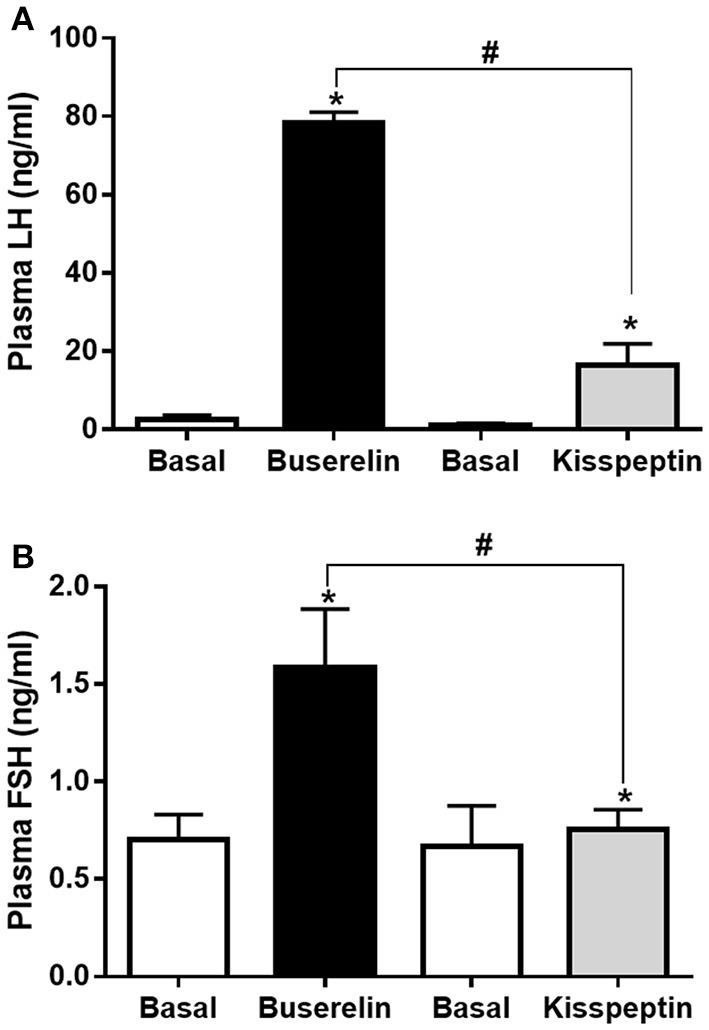
Alteration in the plasma LH and FSH levels in adult Murrah buffaloes by buserelin and kisspeptin. Experimental buffaloes that received either kisspeptin (20 μg/kg body weight, IV) or buserelin (10 μg/animal, IV) on 11th or 8th day of estrous cycle (i.e., correlating with emergence of waves depending on whether the oestrous cycle is of two-wave or three-wave pattern, respectively) were sampled before (basal) and after drug administration (post-drug samples were collected at the peak time-point as observed in pre-pubertal animals: at 15 min after kisspeptin and at 2 h after buserelin). **(A)** While both the drugs increased plasma LH levels (*P < 0.05, *t*-test), the increase was significantly more in buserelin group (*n* = 6) compared to kisspeptin group (*n* = 6) (^#^*P* = 0.02, *t*-test). **(B)** Buserelin significantly increased FSH concentration while kisspeptin did not (*, ^#^*P* < 0.05, *t*-test). Data represented as mean ± SEM.

Experimental buffaloes that received either kisspeptin (20 μg/kg, IV) or buserelin (10 μg, IV) on 11th or 8th day of estrous cycle, i.e., in correlation with emergence of waves, depending on whether the oestrous cycle is of two-wave or three-wave pattern, respectively, were sampled before (basal) and after (at the peak time point as seen from pre-pubertal data: at 15 min after kisspeptin and at 2 h after buserelin) drug administration. While both the drugs increased plasma LH levels (*P* < 0.05, *t*-test), the increase was significantly more in buserelin group compared to kisspeptin group (*P* = 0.02, *t*-test; Figure 5A). In addition, while kisspeptin did not increase plasma FSH concentration compared to basal level, buserelin significantly increased FSH concentration (*P* < 0.05, *t*-test; Figure 5B).

### Effect of kisspeptin vs. buserelin on follicular dynamics in adult buffaloes

There was no significant difference in the length of the oestrous cycle between kisspeptin- and buserelin-treated groups. The number of follicles at wave emergence was significantly more in kisspeptin-treated group (12.16 ± 0.47) compared to buserelin-treated group (10.33 ± 0.42; *P* < 0.05, *t*-test). The average number of follicles in the wave was also significantly higher in kisspeptin-treated group (9.33 ± 0.49) than in buserelin-treated group (7.16 ± 0.30; *p* < 0.05, *t*-test). There was no significant difference between the groups in maximum diameter as well as growth rate of dominant and subordinate follicles. However, the diameter of ovulatory follicle was larger (13.83 ± 0.47 mm) in kisspeptin group compared to buserelin group (11.00 ± 0.36 mm; *P* < 0.05, *t*-test). The results are summarized in Table 2.

**Table 2:** Effect of kisspeptin and buserelin on follicular dynamics in adult Murrah buffaloes.

**S. No**.	**Parameters**	**Kisspeptin (*n* = 6)**	**Buserelin (*n* = 6)**
1.	Length of oestrous cycle (days)	22.66 ± 0.42	24.16 ± 0.30
2.	Number of follicles at wave emergence after drug administration	12.16 ± 0.47^*^	10.33 ± 0.42
3.	Total number of follicles	9.33 ± 0.49^*^	7.16 ± 0.30
4.	Growth rate of the dominant follicle (mm/day)	1.66 ± 0.18	1.31 ± 0.08
5.	Growth rate of the subordinate follicle (mm/day)	1.16 ± 0.14	0.8 ± 0.10
6.	Maximum diameter of ovulatory follicle (mm)	13.83 ± 0.47^*^	11.00 ± 0.36
7.	Maximum diameter of subordinate follicle (mm)	9.23 ± 0.08	8.33 ± 0.33

*Experimental buffaloes that received either kisspeptin (20 μg/kg body weight, IV) or buserelin (10 μg/animal, IV) on 11th or 8th day of estrous cycle (i.e., correlating with emergence of waves depending on whether the oestrous cycle is of two-wave or three-wave pattern, respectively) were observed for follicular dynamics using ultrasound examination on alternate days. Kisspeptin administration significantly increased the number of follicles at wave emergence, total number of follicles and diameter of ovulatory follicle. Data represented as mean ± SEM*.

* *P < 0.05, t-test*.

### Effect of kisspeptin or buserelin on follicular dynamics during oestrus synchronization

All the animals had at least one follicle of more than 10 mm diameter at the time of administration of 1st dose of drugs. The proportion of animals that ovulated after 1st dose of drug (33.33 vs. 16.66%), the proportion of animals that showed onset of a new follicular wave (100 vs. 83.33%), the oestrous response rate (83.33 vs. 66.66%) and conception rate (66.66 vs. 50%) in kisspeptin-PGF_2α_-kisspeptin group *vs*. buserelin-PGF_2α_-buserelin group were statistically insignificant (*P* > 0.05, Fisher's exact test). The number of follicles at wave emergence was significantly higher in kisspeptin group (11.00 ± 0.51) than in buserelin group (8.33 ± 0.49; *P* < 0.05, *t*-test). In addition, the mean diameter of ovulatory follicle after 2nd dose of drug was also significantly higher in kisspeptin group (15.58 ± 0.42 mm) compared to buserelin group (13.6 ± 0.47 mm; *P* < 0.05, *t*-test). Further, the duration of oestrus was also higher in kisspeptin group than in buserelin group (22.5 ± 1.4 h vs. 18.16 ± 1.01 h; *P* < 0.05, *t*-test). Follicular dynamics observed during oestrous synchronization in buffaloes with kisspeptin or buserelin is shown in Table 3.

**Table 3:** Follicular dynamics during oestrus synchronization: Effect of two different protocols involving kisspeptin or buserelin.

**S. No**.	**Parameters**	**Kisspeptin (*n* = 6)**	**Buserelin (*n* = 6)**
1.	Proportion of animals that ovulated after first dose	33.33 (2/6)	16.66 (1/6)
2.	Proportion of animals that started new follicular wave after first dose	100 (6/6)	83.33(5/6)
3.	Time of emergence of new follicular wave since drug (h)	52.00 ± 1.31	50.5 ± 1.1
4.	Number of follicles at wave emergence	11.00 ± 0.51^*^	8.33 ± 0.49
5.	Maximum diameter of ovulatory follicle after second dose	15.58 ± 0.42^*^	13.6 ± 0.47
6.	Proportion of animals that ovulated after second dose	83.33 (5/6)	83.33 (5/6)
7	Oestrous response	83.33(5/6)	66.66 (3/6)
8.	Duration of oestrus (h)	22.5 ± 1.4^*^	18.16 ± 1.01
9.	Conception rate on day 45 since FTAI (%)	66.66 (4/6)	50.00 (3/6)

Ovarian ultrasonography was carried out daily from the day of initiation of the synchronization protocol, using either kisspeptin or buserelin, until insemination. Number of follicles at wave emergence, maximum diameter of ovulatory follicle and duration of oestrus were greater in kisspeptin group. Data represented as mean ± SEM. *P < 0.05, t-test.

## Discussion

Buserelin, as expected, resulted in a robust and sustained release of LH. Though kisspeptin dose-dependently increased plasma LH, the release was short-lived and was smaller compared to buserelin-induced LH release. This pattern of immediate release of LH and ensuing declination after the initial rise have been observed earlier with kisspeptin administration (10). While buserelin sustained the FSH release, kisspeptin had little effect on plasma FSH. Less significant FSH release, as compared to LH release, upon kisspeptin administration has been reported earlier in cattle, pigs and mice (8, 11, 12). FSH release is not fully controlled by GnRH as ovarian factors like activin and inhibin are also involved in FSH regulation (13, 14). Hence, high FSH levels for several hours upon peripheral administration of kisspeptin observed elsewhere in ovariectomized oestradiol treated ewes may be due to lack of inhibin upon ovariectomy (10).

Both IV and IM injections of kisspeptin were similar in terms of duration of LH increase, however, IV injection resulted in an earlier increase and a higher LH concentration than the IM injection. This important finding may be of significant commercial and clinical importance. The peak LH values were similar with both 10 and 20 μg/kg dose rates of kisspeptin, IV. Hence, we chose to inject kisspeptin at the smallest dose tested that gives near-maximal response, i.e., 10 μg/kg, IV, repeatedly, so as to simulate injecting a long-acting form of kisspeptin and study the LH release response. Interestingly, LH response to repeated injections of kisspeptin were similar to the response observed upon single injection of buserelin, a long-acting GnRH agonist, however, there was no evidence of additive effect. A single dose of kisspeptin inducing a short-lived stimulation of LH release and repeated intravenous administration of kisspeptin inducing a sustained train of FSH and LH pulses were reported earlier in livestock (10, 15). Lack of additive effect upon repeated kisspeptin has been shown earlier in pre-pubertal ewes (16). Pulsatile administration of kisspeptin once every hour for 24 h has been reported to enhance gonadotropin secretion and ovarian steroidogenesis and to stimulate LH surge and ovulation (17). However, we did not study the follicular dynamics upon repeated kisspeptin administration.

Continuous infusion of human metastin 45–54 has been shown to desensitize GPR54 in agonadal juvenile male rhesus monkeys (18). Once-daily subcutaneous injection as well as continuous administration of kisspeptin analogs, TAK-448 and TAK-683, suppressed the pituitary-gonadal functions in male rats as evidenced by reduction in testosterone levels (19). On the contrary, the unstinted LH response upon repeated kisspeptin, observed in our study, suggests lack of receptor desensitization. It has been observed that the peak LH level in buffaloes during LH surge is 20-40 ng/ml and the surge lasts for 7–12 h (20). Though similar LH levels are reached with kisspeptin in our study, the duration of release is less compared to endogenous LH surge requiring further studies on receptor desensitization in buffaloes.

In pre-pubertal buffaloes, we observed that the number of follicles were significantly more in buserelin group, while the size of follicles was larger in kisspeptin group. Hence, it appears that kisspeptin and buserelin differentially govern follicular dynamics. Presence of kisspeptin and GnRH receptors have been shown in the ovarian cells (4, 21, 22). Hence, systemic injections of these drugs possibly act via local ovarian mechanisms, in addition to the effects via release of gonadotropins that act on ovarian cells. Buserelin-induced increase in FSH may have contributed for greater increase in number of follicles in buserelin group. An earlier report of effect of kisspeptin on follicular dynamics observed that kisspeptin-53 at 2 nmol/kg increased the size of dominant follicle in cattle (23). However, no effects on number of follicles were observed or reported so far and, the mechanism behind differential regulation of follicular dynamics by kisspeptin and buserelin remains unexplored.

Our further studies on gonadotropin and follicular responses upon kisspeptin and buserelin administration in adult buffaloes were carried out when the drugs were administered at the beginning of the last follicular wave of oestrous cycle that leads to ovulation. In adult buffaloes, similar to pre-pubertal heifers, buserelin-induced LH response was many folds higher compared to kisspeptin-induced response. However, in contrast to heifers, average number of follicles in a wave and number of follicles at wave emergence were higher in kisspeptin group compared to buserelin group. The diameter of the ovulatory follicle was also larger in kisspeptin group. This was observed again in the adult buffaloes when the drugs were administered randomly for oestrous synchronization. This suggests that differential regulation of follicular dynamics kisspeptin and GnRH may further differ depending on the cyclicity or reproductive age of the animals. Differential LH responses to kisspeptin administration at different time points within oestrous cycle have been reported earlier in ewes (24). However, ours is the first study to report such responses among different age-groups. Age-dependent variation in the responses might possibly be due to differences in the maturation of the reproductive axis and neuroendocrine signaling, oestrous cycle, body weight, etc. (25). Elucidating the mechanism behind this variation was beyond the scope of our study.

Though we observed promising results with kisspeptin when we used it for oestrous synchronization, unless the studies are repeated in a large number of animals, we may not be able to confirm the results. As pregnancy rate is the ultimate parameter to assess the success of a synchronization protocol, the study has to be carried out in a larger herd to arrive at a conclusion. However, ovulatory rates of more than 80% induced by kisspeptin, a short-acting peptide, similar to that induced by buserelin, a long-acting GnRH agonist, is intriguing. Kisspeptin treatment resulting in ovulation has been reported earlier in ewes and cows (10, 23). However, our study is the first one to report the same in comparison with a routinely used GnRH agonist.

Though this study is of important clinical relevance, the study limitations, such as wide variation in the age of adult experimental buffaloes used, seasonal variation in hormonal levels (i.e., pre-pubertal buffaloes were studied during summer while adult buffaloes were studied during fall and winter), and usage of bovine ELISA kits for analyzing LH and FSH concentration in plasma samples of experimental buffaloes, are to be acknowledged and accordingly, the results may be cautiously interpreted.

Whether kisspeptin can be administered at larger dose rates, i.e., more than 20 μg/kg, the maximal dose used in our study, so as to enhance the LH response, needs to be tested further. Optimization of the effective dose based on LH release and on follicular dynamics will help us to formulate an oestrus synchronization regimen that can be used as a treatment strategy for repeat breeders and also as a management strategy for embryo collection and transfer in embryo biotechnology. This will also help us to develop a kisspeptin-based protocol to induce oestrus and ovulation in pre-pubertal and anoestrus animals.

Novel therapeutic strategies are warranted for treating reproductive disorders in farm animals. This project, aiming at studying the effect of kisspeptin on plasma endocrine profile and follicular dynamics, enables us to compute kisspeptin-based treatment strategies for fertility disorders in livestock. This study supplements the potential applications of kisspeptin in reproductive management of farm animals that has been thoroughly reviewed Caraty et al. (26).

In conclusion, this study finds that IV administration of kisspeptin might be more beneficial in terms of LH release rather than IM. It also observes that kisspeptin and GnRH may differentially regulate follicular dynamics depending on reproductive age of the animals. The mechanism for this differential regulation needs to be explored. A novel protocol using kisspeptin for oestrus synchronization tested in adult cycling buffaloes demonstrated beneficial effects, however, a larger sample size is required to further confirm the results.

## Author contributions

VP performed follicular dynamics, assisted in blood sampling, compiled and analyzed the data. SR collected blood samples from pre-pubertal animals, performed ELISA, biochemical tests and FACS, and compiled data. CR provided the necessary infrastructure at the Department of Veterinary Gynaecology and Obstetrics, College of Veterinary Science, PVNR TVU. AG planned follicular dynamics experiments, analyzed and interpreted the data, and prepared tables. KA collected blood samples from adult animals, performed ELISA and biochemical tests, and compiled data. SB assisted in performing follicular dynamics, sample collection and compilation of data. SS assisted in plasma and serum separation, ELISA and biochemical tests. SK assisted in performing follicular dynamics and compilation of data. SV conceived and planned experiments, analyzed and interpreted the data, wrote the manuscript and prepared the figures.

### Conflict of interest statement

The authors declare that the research was conducted in the absence of any commercial or financial relationships that could be construed as a potential conflict of interest.
